# Supplementary material for: Environmental selection overturns the decay relationship of soil prokaryotic community over geographic distance across grassland biotas
Source: eLife. 2022 Jan 24;11:e70164. doi: 10.7554/eLife.70164 (PMC8828049; doi:10.7554/eLife.70164)
Supplement: Supplementary file 1. [file elife-70164-supp1.docx]

**Supplementary file 1** Mantel test between the prokaryotic community similarity based on Bray-Curtis index and environment variables

|  | All | | | | Alpine | | | | Temperate | | | | Alpine × Temperate | | | |
| --- | --- | --- | --- | --- | --- | --- | --- | --- | --- | --- | --- | --- | --- | --- | --- | --- |
|  | Topsoil | | Subsoil | | Topsoil | | Subsoil | | Topsoil | | Subsoil | | Topsoil | | Subsoil | |
|  | r | p | r | p | r | p | r | p | r | p | r | p | r | p | r | p |
| MAP | 0.276 | **<0.001** | 0.259 | **<0.001** | 0.274 | **<0.001** | 0.280 | **<0.001** | 0.331 | **<0.001** | -0.006 | 0.797 | 0.157 | **<0.001** | 0.128 | **<0.001** |
| MAT | -0.027 | **0.013** | -0.008 | 0.441 | 0.022 | 0.343 | -0.099 | **<0.001** | -0.063 | **0.004** | -0.035 | 0.109 | -0.039 | **0.013** | -0.006 | **0.694** |
| pH | 0.137 | **<0.001** | 0.094 | **<0.001** | 0.253 | **<0.001** | 0.204 | **<0.001** | 0.053 | **0.016** | 0.023 | 0.289 | -0.042 | 0.006 | -0.027 | 0.084 |
| SOC | 0.274 | **<0.001** | 0.191 | **<0.001** | 0.420 | **<0.001** | 0.277 | **<0.001** | 0.124 | **<0.001** | 0.067 | **0.002** | 0.187 | **<0.001** | 0.148 | **<0.001** |
| TN | 0.265 | **<0.001** | 0.170 | **<0.001** | 0.387 | **<0.001** | 0.254 | **<0.001** | 0.115 | **<0.001** | 0.060 | 0.006 | 0.193 | **<0.001** | 0.137 | **<0.001** |
| TP | 0.028 | **<0.001** | 0.030 | **0.006** | -0.000 | 0.985 | 0.027 | 0.240 | 0.118 | **<0.001** | 0.094 | **<0.001** | -0.146 | **<0.001** | -0.124 | **<0.001** |
| Long-term environment variables | 0.337 | **0.001** | 0.291 | **0.001** | 0.363 | **0.001** | 0.322 | 0.001 | 0.328 | **0.001** | 0.002 | **0.447** | 0.181 | **0.002** | 0.154 | **0.006** |
| SWC | 0.353 | **<0.001** | 0.332 | **<0.001** | 0.602 | **<0.001** | 0.553 | **<0.001** | 0.093 | **<0.001** | 0.003 | 0.909 | 0.264 | **<0.001** | 0.241 | **<0.001** |
| AP | 0.185 | **<0.001** | 0.074 | **<0.001** | 0.355 | **<0.001** | 0.145 | **<0.001** | 0.002 | 0.912 | 0.080 | **<0.001** | 0.197 | **<0.001** | 0.080 | **<0.001** |
| DOC | 0.230 | **<0.001** | 0.145 | **<0.001** | 0.350 | **<0.001** | 0.279 | **<0.001** | 0.052 | **0.017** | 0.002 | 0.913 | 0.195 | **<0.001** | 0.088 | **<0.001** |
| DON | 0.397 | **<0.001** | 0.330 | **<0.001** | 0.334 | **<0.001** | 0.260 | **<0.001** | 0.073 | **<0.001** | 0.035 | 0.116 | 0.199 | **<0.001** | 0.159 | **<0.001** |
| NH_4_^+^ | 0.217 | **<0.001** | 0.109 | **<0.001** | 0.284 | **<0.001** | 0.077 | **<0.001** | 0.018 | 0.408 | 0.026 | 0.236 | 0.109 | **<0.001** | 0.092 | **<0.001** |
| NO_3_^-^ | 0.222 | **<0.001** | 0.111 | **<0.001** | 0.121 | **<0.001** | 0.078 | **<0.001** | 0.048 | **0.027** | 0.053 | **0.016** | 0.008 | 0.611 | -0.002 | 0.920 |
| Short-term environment variables | 0.391 | **0.001** | 0.294 | **0.001** | 0.367 | **0.001** | 0.305 | **0.001** | 0.104 | 0.091 | 0.039 | **0.251** | 0.258 | **0.001** | 0.216 | **0.001** |
| Latitude | 0.094 | **<0.001** | 0.091 | **<0.001** | 0.257 | **<0.001** | 0.250 | **<0.001** | 0.132 | **<0.001** | 0.094 | **<0.001** | -0.436 | **<0.001** | -0.360 | **<0.001** |
| Longitude | 0.136 | **<0.001** | 0.124 | **<0.001** | 0.124 | **<0.001** | 0.092 | **<0.001** | 0.431 | **<0.001** | 0.353 | **<0.001** | -0.419 | **<0.001** | -0.350 | **<0.001** |
| Altitude | 0.178 | **<0.001** | 0.158 | **<0.001** | 0.385 | **<0.001** | 0.445 | **<0.001** | 0.352 | **<0.001** | 0.253 | **<0.001** | -0.469 | **<0.001** | -0.442 | **<0.001** |
| Distance* | -0.124 | **<0.001** | -0.094 | **<0.001** | -0.183 | **<0.001** | -0.150 | **<0.001** | -0.359 | **<0.001** | 0.005 | 0.816 | 0.446 | **<0.001** | 0.366 | **<0.001** |
| Geographic variables | 0.140 | **<0.001** | 0.130 | **<0.001** | 0.347 | **<0.001** | 0.338 | **<0.001** | 0.322 | **<0.001** | 0.256 | **<0.001** | -0486 | **<0.001** | -0.408 | **<0.001** |

***Calculated by the geographic distance for paired samples based on the longitude and latitude of each sample**
