# Supplementary material for: Environmental selection overturns the decay relationship of soil prokaryotic community over geographic distance across grassland biotas
Source: eLife. 2022 Jan 24;11:e70164. doi: 10.7554/eLife.70164 (PMC8828049; doi:10.7554/eLife.70164)
Supplement: Supplementary file 2. [file elife-70164-supp2.docx]

**Supplement file 2** Partial Mantel test between prokaryotic community similarity based on Bray-Curtis index and environmental variables.

|  | All | | | | Alpine | | | | Temperate | | | | Alpine × Temperate | | | | |
| --- | --- | --- | --- | --- | --- | --- | --- | --- | --- | --- | --- | --- | --- | --- | --- | --- | --- |
|  | Topsoil | | Subsoil | | Topsoil | | Subsoil | | Topsoil | | Subsoil | | Topsoil | | Subsoil | | |
|  | r | p | r | p | r | p | r | p | r | p | r | p | r | p | r | p |  |
| MAP | 0.185 | **0.001** | 0.168 | **0.002** | 0.235 | **0.002** | 0.199 | **0.007** | 0.310 | **0.001** | 0.316 | **0.001** | 0.060 | 0.101 | 0.047 | 0.165 |  |
| MAT | -0.054 | 0.869 | -0.067 | 0.922 | 0.065 | 0.122 | -0.027 | 0.640 | -0.012 | 0.574 | 0.040 | 0.230 | 0.000 | 0.498 | -0.021 | 0.625 |  |
| pH | 0.014 | 0.367 | 0.044 | 0.160 | 0.053 | 0.223 | 0.143 | 0.053 | -0.101 | 0.952 | -0.174 | 0.998 | -0.001 | 0.516 | -0.004 | 0.542 |  |
| SOC | 0.061 | 0.154 | -0.039 | 0.648 | 0.054 | 0.279 | -0.166 | 0.990 | -0.197 | 1.000 | -0.097 | 0.891 | 0.231 | **0.001** | 0.120 | **0.010** |  |
| TN | 0.095 | 0.084 | 0.004 | 0.380 | 0.075 | 0.166 | 0.060 | 0.230 | 0.004 | 0.435 | -0.135 | 0.991 | 0.151 | **0.003** | 0.046 | 0.146 |  |
| TP | -0.022 | 0.642 | 0.016 | 0.360 | 0.074 | 0.074 | 0.095 | 0.065 | -0.081 | 0.873 | 0.012 | 0.383 | -0.143 | 0.999 | -0.072 | 0.908 |  |
| Long-term environment variables | 0.149 | **0.016** | 0.063 | 0.165 | -0.019 | 0.547 | -0.158 | 0.994 | -0.267 | 1.000 | -0.217 | 1.000 | 0.121 | **0.010** | 0.049 | 0.174 |  |
| SWC | 0.100 | 0.080 | 0.001 | 0.425 | 0.160 | 0.062 | -0.072 | 0.743 | -0.125 | 0.954 | -0.046 | 0.638 | 0.112 | **0.015** | 0.094 | **0.032** |  |
| AP | 0.019 | 0.327 | -0.068 | 0.849 | -0.027 | 0.541 | -0.119 | 0.933 | -0.099 | 0.919 | -0.053 | 0.720 | 0.003 | 0.387 | 0.064 | 0.124 |  |
| DOC | 0.198 | **0.018** | 0.091 | 0.124 | 0.103 | 0.140 | -0.078 | 0.790 | -0.127 | 0.981 | -0.165 | 0.999 | 0.307 | **0.001** | 0.206 | **0.001** |  |
| DON | 0.143 | **0.040** | 0.337 | **0.001** | -0.019 | 0.478 | 0.211 | **0.029** | -0.130 | 0.978 | -0.136 | 0.989 | 0.398 | **0.001** | 0.401 | **0.001** |  |
| NH_4_^+^ | 0.068 | 0.137 | -0.011 | 0.480 | -0.158 | 0.996 | -0.130 | 0.952 | -0.107 | 0.934 | -0.024 | 0.570 | 0.223 | **0.001** | 0.023 | 0.350 |  |
| NO_3_^-^ | 0.062 | 0.174 | 0.034 | 0.258 | 0.023 | 0.347 | -0.075 | 0.824 | 0.079 | 0.131 | -0.040 | 0.691 | -0.135 | 0.990 | 0.040 | 0.234 |  |
| Short-term environment variables | 0.253 | **0.002** | 0.216 | **0.009** | 0.150 | 0.059 | 0.010 | 0.450 | -0.072 | 0.736 | -0.198 | 0.999 | 0.523 | **0.001** | 0.465 | **0.001** |  |
| Latitude | -0.020 | 0.736 | 0.009 | 0.390 | 0.183 | **0.001** | 0.224 | **0.001** | 0.062 | 0.163 | 0.068 | 0.171 | -0.541 | 1.000 | -0.433 | 1.000 |  |
| Longitude | 0.060 | **0.029** | 0.066 | **0.022** | 0.019 | 0.297 | 0.035 | 0.215 | 0.412 | **0.001** | 0.353 | **0.001** | -0.525 | 1.000 | -0.430 | 1.000 |  |
| Altitude | 0.079 | **0.001** | 0.086 | **0.001** | 0.391 | **0.001** | 0.430 | **0.001** | 0.338 | **0.001** | 0.244 | **0.001** | -0.672 | 1.000 | -0.588 | 1.000 |  |
| Distance* | 0.055 | **0.036** | 0.063 | 0.114 | 0.164 | **0.001** | 0.183 | **0.001** | 0.325 | **0.001** | 0.285 | **0.001** | -0.544 | 1.000 | -0.438 | 1.000 |  |
| Geographic variables | 0.122 | **0.001** | 0.114 | **0.001** | 0.364 | **0.001** | 0.370 | **0.001** | 0.380 | **0.001** | 0.306 | **0.001** | -0.5664 | 1.000 | -0.462 | 1.000 |  |

***Calculated by the geographic distance for paired samples based on the longitude and latitude of each sample**
